# Assessment of antimicrobial and anthelmintic activity of silver nanoparticles bio-synthesized from *Viscum orientale* leaf extract

**DOI:** 10.1186/s12906-023-03982-1

**Published:** 2023-05-22

**Authors:** Dugganaboyana Guru Kumar, Raghu Ram Achar, Jajur Ramanna Kumar, Ganamaedi Amala, Velliyur Kanniappan Gopalakrishnan, Sushma Pradeep, Ali A. Shati, Mohammad Y. Alfaifi, Serag Eldin I. Elbehairi, Ekaterina Silina, Victor Stupin, Natalia Manturova, Chandan Shivamallu, Shiva Prasad Kollur

**Affiliations:** 1 Division of Biochemistry, School of Life Sciences, JSS Academy of Higher Education and Research (Deemed to Be University), Sri Shivarathreeshwara Nagara, Mysuru, 570015 Karnataka India; 2 School of Medicine, Bule Hora University Institute of Health, Bule Hora University, Bule Hora, Ethiopia; 3 Department of Biotechnology and Bioinformatics, JSS Academy of Higher Education, Mysore, India; 4 Biology Department, Faculty of Sciences, King Khalid University, Abha, Saudi Arabia; 5 Department of Hospital Surgery, N.I. Pirogov Russian National Research Medical University (RNRMU), Moscow, 117 997 Russia; 6 School of Physical Sciences, Amrita Vishwa Vidyapeetham, Mysuru Campus, Mysuru, 570 026 Karnataka India

**Keywords:** Silver nanoparticles, *Viscum orientale*, Antihelmenthic, Hemagglutination, Anti-oxidation, Antimicrobial

## Abstract

**Background:**

*Viscum orientale* is a largely used parasitic plant with traditional medicinal properties. They are considered to possess the medicinal properties of host tree which they grow on. It’s a least explored plant with ethanopharmacological importance. As a result, the current work aimed to investigate the biological effects of *Viscum orientale* extract and silver nanoparticles (AgNPs) generated from it.

**Methods:**

AgNPs synthesized using *Viscum orientale plant* extract and analysed on time dependent series and was characterized using Ultra Violet UV–visible spectra, Fourier Transform Infrared Spectroscopy FTIR, X-ray diffraction (XRD), Energy Dispersive X-ray Spectroscopy (EDX), Scanning Electron Microscopy (SEM). Further using disc method anti-microbial assay was performed following antioxidation screening using 1,1-diphenyl-2-picryl-hydrazyl (DPPH), reducing power and nitric oxide content and heamgglutination with human blood.

**Results:**

On green synthesis using silver, the phyto contituents of plant *Viscum orientale* effectively reduced silver ions at 3-4 h of continuous stirring to form AgNPs. UV–vis spectra showed a typical peak of AgNPs at 480 nm. The FTIR analysis confirmed the covering of silver layers to bio-compounds of the extract. SEM analysis represented AgNPs as spherical morphologies ranging from 119–222 nm. AgNPs exhibited impressive zone of inhibition against *Escherichia coli* (8.1 ± 0.3 mm), *Staphylococcus aureus* (10.3 ± 0.3 mm), *Bacillus subtilis* (7.3 ± 0.3 mm), *Bacillus cereus* (8.2 ± 0.3 mm), *Salmonella typhi* (7.1 ± 0.2 mm). AgNps exhibited efficiency against DPPH at EC_50_ value of 57.60 µg/ml. and reducing power at EC_50_ of 53.42 µg/ml and nitric oxide scavenging of EC_50_ of 56.01 µg/ml concentration. Further, anthelmintic activity results showed synthesized nanoparticles significant reduction in the paralysis time to 5.4 ± 0.3 min and death time to 6.5 ± 0.6 min in contrast to the individual factors. On hemagglutination using AgNPs, above 80 µg/ml of concentration showed very significant effect on comparison with water extract.

**Conclusion:**

Synthesized AgNPs using *Viscum orientale* water extract displayed versatile biological activity than individual extract. This study has forecasted a new path to explore more on this AgNPs for further research.

**Supplementary Information:**

The online version contains supplementary material available at 10.1186/s12906-023-03982-1.

## Background

Nanotechnology is a relatively recent discipline of science that offers a diverse set of uses [1]. Recent years its gaining importance in the field of biology and biomedical sciences. Because of its nano size it’s been efficiently correlated with the biological components in the system and have promising effects [2, 3]. Majorly green synthesis of nanoparticles by using silver metal is being more advantageous because of its biocompatibility and physicochemical properties [4]. Due to the toxic effects of chemically synthesized nanoparticles, green synthesis using the plant secondary metabolites for nanoparticle synthesis is gaining importance [5]. Plant extracts with huge range of unique phytochemicals are being contributed in the reduction of silver ions and assists in nanoparticle formation [6]. Green synthesis is a very cost effective and eco-friendly and non-toxic to the cells [7]. Henceforth green synthesis is being widely accepted technique in the field of medicine. Many previous reports have demonstrated the bioactivities of AgNPs namely in the field of cancer, against oxidative stress, apoptosis. Many plant resources are screened for its effectiveness biologically in its synthesized AgNP forms [5].

*Viscum orientale* is a parasitic plant and has been least explored [8]. *Viscum orientale* Willd. (dyer’s oleander mistletoe) is largely used in traditional medicines and is believed to derive some particular property from its host tree [8]. It’s a commonly found species in India. Even with its minute toxicity effects, it has proven medicinal properties from ancient time namely, anti-microbial, substitute for nux-vomica, Poultice of leaves is used for neuralgia; ashes of the plant for the treatment of skin diseases [9]. It’s also used to treat giddiness, pustules and stiffness. Recent studies have reported the in vivo anti-nociceptive and reinforced CNS (Central Nervous System) depressant activities [8]. They have even reported the presence of five major polyphenols in the leaf namely, vanillic acid, quercetin, ellagic acid, gallic acid and, caffeic acid Hence in the present study we have explored the possibly applicable biological effects of synthesized nanoparticles using *Viscum orientale* water extract.

## Materials and methods

### Collection of plant material

*Viscum orientale* was collected from the University of Mysore campus (12.3081° N Latitude, 76.6390° E Longitude), Mysuru, Karnataka. The tree was authenticated by Prof. Shivalingaiah, Department of Botany, Maharani’s Science College for Women, Mysore, Karnataka, and the herbarium was deposited in Department of Biotechnology and Bioinformatics, JSS AHER, Mysore, Karnataka, India with voucher number VO15. The leaves of the plant were collected, washed and shade dried for one week. A minimum of one week of shade drying was necessary as the leaves were collected in the month of December-January. Dried material was powdered thoroughly for further extraction.

### Chemicals

The reagents employed in the experiment were all analytical grades. DPPH (sigma chemicals) Silver Nitrate (MERCK, Germany), all the chemicals and reagents were procured from padmashri chemicals. Mysuru.

### Preparation of plant extracts

Dried samples were extracted continuously using range of organic solvents from non-polarity to polarity (Hexane > Chloroform > Ethylacetate > acetone > Water) and the samples were named as VOHE, VOCE, VOEA, VOAE and VOWE. The extraction was performed by overnight stirring at room temperature and was repeated in each solvent till the sample becomes colorless. Extracted solvents were dried using rotary evaporator and lyophilized and stored at 4^0^C, till further use.

### Phytochemical analysis

Plant extracts were assessed for the existence of different phytochemicals namely, flavonoids, alkaloids, saponins, phenols, carbohydrates, glycosides, phytosterols, proteins and terpinoids using standard method [8].

### Synthesis of silver nanoparticles

Synthesis of silver nanoparticles at varying stirring time intervals [10]. In a 250ml beaker, 1mM of 100ml Silver nitrate solution was prepared. 10-15ml of leaf extract was added dropwise to the silver nitrate solution with vigorous shaking. The bio-reduced silver nanoparticles solution was run on a UV-Visible spectrophotometer at regular time intervals (0, 1, 2, 3, 4 hours) to evaluate the influence of stirring time on silver nanoparticles formation. Then the graph is plotted by λ_max_ on x-axis and absorbance on y-axis. The synthesized AgNPs were lyophilized, weighed and stored for further use.

### Characterization of synthesized silver nanoparticle

The reduced form of silver on synthesis of nanoparticles were monitored by UV–visible spectra with the range of 200–800 nm, using a UV–Vis spectrophotometer Shimadzu-UV-1800 (Tokyo, Japan) with distilled water as a reference. In the wavelength range 4000–400 cm^−1^, FTIR spectra were recorded using a Perkin Elmer FTIR RX1 (Dresden, Germany). XPERT-PRO (Bristol, UK)used monochromatic Cu radiation (k = 1.5406 A °) at 40 kV and 30 mA in a 2 h angle pattern for the X-ray diffraction (XRD) examination. Scanning was done between 208 and 808. The crystalline structure was compared to the photographs. FESEM outfitted with an EDAX attachment was used to perform EDAX analysis of silver nanoparticles on a SUPRA55 (CARL ZEISS, Germany). The morphology, size, and shape of the silver nanoparticles were determined via SEM examination. HITACHI H-800 (Tokyo, Japan) SEM tests were performed at 200 kV. A drop of the bio-reduced diluted solution was placed on a carbon-coated copper grid and dried under a lamp to create the SEM grid. Malvern instruments were used to measure the size distribution and stability of AgNPs, as well as DLS and zeta potential [11].

### Antioxidant activity

#### Diphenyl-2-picrylhydrazyl radical scavenging activity

Blois (1958) method was used to assess the AgNPs 1,1-diphenyl-2-picryl-hydrazyl (DPPH) free radical scavenging capacity [12]. In several test tubes, different concentrations (20, 40, 60, 80, and 100 μg/ml) of AgNPs and standard butylated hydroxytoluene (BHT) were used. To the above samples, 1 ml of freshly prepared DPPH (0.1 mM) was added and vortexed thoroughly and incubated in dark 30 min. At 517 nm, the absorbance of stable DPPH was measured. As a control, the DPPH without sample was preserved. The inhibition % was used to measure the free radical scavenging activity. The percentage of inhibition was computed, and BHT was used as a reference standard. The percentage inhibition vs. concentration was plotted, and the IC_50_ value was calculated as the concentration required to inhibit radicals by 50%.

### Reducing power capacity

2.5 ml of sodium phosphate buffer was added to the various concentrations of the AgNPs (20, 40, 60, 80, and 100 μg/ml), followed by 2.5 ml of % potassium ferricyanide solution. The content was vortexed well before being incubated for 20 min at 500 °C. Following the incubation period, 2.5 ml of 10% TCA was added to each tube and centrifuged at 3000 g for 10 min. To 5 ml of the supernatant, 5 ml of deionized water was added followed by addition of 1 ml of 1% Ferric chloride and incubated at 35^0^C for 10 min. Absorbance was read at 700 nm. The reference standard was butylated hydroxyl toluene (BHT). The percentage inhibition vs. concentration was plotted, and the IC_50_ value (concentration required for 50% radical inhibition) was calculated [13].

#### Nitric oxide radical scavenging assay

Garrat et al. (1964) developed a nitric oxide radical scavenging test [14]. Briefly, 1 ml of 10 mM Sodium nitroprusside was added to all the tubes containing varied quantities of AgNPs (20, 40, 60, 80, and 100 μg/ml), followed by 1 ml of Griess reagent. The reaction mixture was vortexed well and incubated for 1 h at room temperature. In dispersed light, a pink-colored chromophore form. At 540 nm, the absorbance of the mixture was compared to that of the matching blank solutions. The reference standard was butylated hydroxyl toluene (BHT).

### Antimicrobial activity

#### Preparation of the bacterial sub-culture

The different bacterial culture used for present investigation namely *Escherichia coli, Bacillus cereus, Bacillus subtilis*, *Staphylococcus aureus* and *Salmonella typhi* and were obtained from P.G. Department of Microbiology, Manasagangotri Campus, University of Mysore, Mysuru. To obtain a bacterial subculture, 100 μl of the bacterial culture was combined with 5 ml of sterile nutrient broth and incubated at 37 °C for 16 h [15].

#### Antibacterial activity

Using the agar disc diffusion assay method, the antimicrobial activity of the produced silver nanoparticles was assessed. Briefly, autoclaved nutrient agar plates were prepared and to that test bacterial cultures of 100 μl were then swabbed on the surface. Disc diffusion assay was carried out using standard protocol. An antibiotic, Gentamicin is taken as positive control. 10 μg/ml of Gentamicin, 10 μg/ml, 15 μg/ml and 20 μg/ml of the synthesized silver nanoparticles was applied on separate sterile discs of diameter 6 mm (whatman filter paper discs) and allowed to dry before being placed on the agar medium. The plates were incubated at 37^0^C for 24 h and the resulting zone of inhibition was measured [15].

### Anthelmintic assay

The anthelmintic assay was performed using the Ajaiyeoba et al., (2001) method [16]. Earthworms (6–9 cm) from the *Pheretima posthuma* species were collected for this study due to their relatedness and similarities with the human intestinal roundworms. Earthworms were collected from the soil of moist and wet regions of Mysuru farmlands. (https://bmccomplementmedtherapies.biomedcentral.com/articles/10.1186/s12906-016-1219-5).

The collected earthworms were washed with distilled water to remove the fecal matter [17]. Three groups were made with 3 earthworms in each. The time it took to become paralyzed and die was measured. When there is no visible movement of the worms except when they are shaking hard, it is said to be paralyzed. The worms' death time, on the other hand, was recorded when it was confirmed that they did not move when shaken vigorously or dipped in warm water (50 °C).

### Hemagglutination assay

In Brief, using microtiter plate, different concentrations of synthesized silver nanoparticles (100 μl) was mixed with 100 μl of 2% suspension of human erythrocyte in phosphate buffer (pH 7.2). The plates were left undisturbed for 1 h for agglutination to take place at room temperature. After incubation time, the results were observed visually [18, 19].

### Statistical analysis

Statistical significance was calculated between the groups using two way ANOVA with Turkey’s multiple comparison test. The data was given as a mean ± standard error of the mean (*n* = 3). Graphpad prism 8.0 was used for statistical analysis.

## Results and discussion

### Extraction of different phytochemicals from *Viscum orientale* leaf sample

Plant phytochemicals are responsible for any bioactivity of the plants. Hence for further assessment of the bioactivities, thorough extraction of bioactive components is a foremost step. Therefore, the successive extraction of the leaf sample was performed successively using range of organic solvents based on their polarity. The phyto-components were separated according to the polarity.

On calculating the yield of the different solvent extracts, Ethyl acetate (13.4%) and water (12.25%) extract gave high percent of yield in comparison with hexane (5.2%), chloroform (8.9%) and acetone (2.1%) (Table 1). Further on qualitative analysis for presence of different phytochemicals groups, revealed the presence of diverse group of phyto-chemical in water extract than other solvent extract (Table 2). This confirmed the water extract with huge collection of phytochemicals responsible for the bioactivity. Similarly previous studies have also used polar solvent extract for screening the specific biological activity of interest using *Viscum orientale* leaf sample (10). Henceforth, for green synthesis of silver nanoparticles (AgNPs), water extract was selected.

**Table 1:** Percentage yield of different solvent extracts of *Viscum orientale* leaf

Solvent extract	Abbreviation	Yield (%) g/100 g of leaf extract
Hexane	VOLHE	5.2
Chloroform	VOLCE	8.9
Ethyl acetate	VOLEA	13.4
Acetone	VOLAE	2.1
Water	VOLWE	12.25

**Table 2:** Screening for different phytochemical constituents in *Viscum orientale* leaf extracts

Constituents	VOLHE	VOLCE	VOLEA	VOLAE	VOLWE
Alkaloids	-	+	+	+	+
Saponins	+	-	-	-	-
Flavonoids	-	-	+	-	+
Phenols and tannins	-	+	+	+	+
Carbohydrates	-	-	-	-	+
Glycosides	-	-	-	-	-
Phytosterols	+	-	-	+	+
Protein and amino acid	-	-	-	-	+
Terpenoids	-	-	-	+	+

‘ + ’ indicates presence and ‘-’ indicates absence

### Synthesis of nanoparticles

**Fig. 1:**
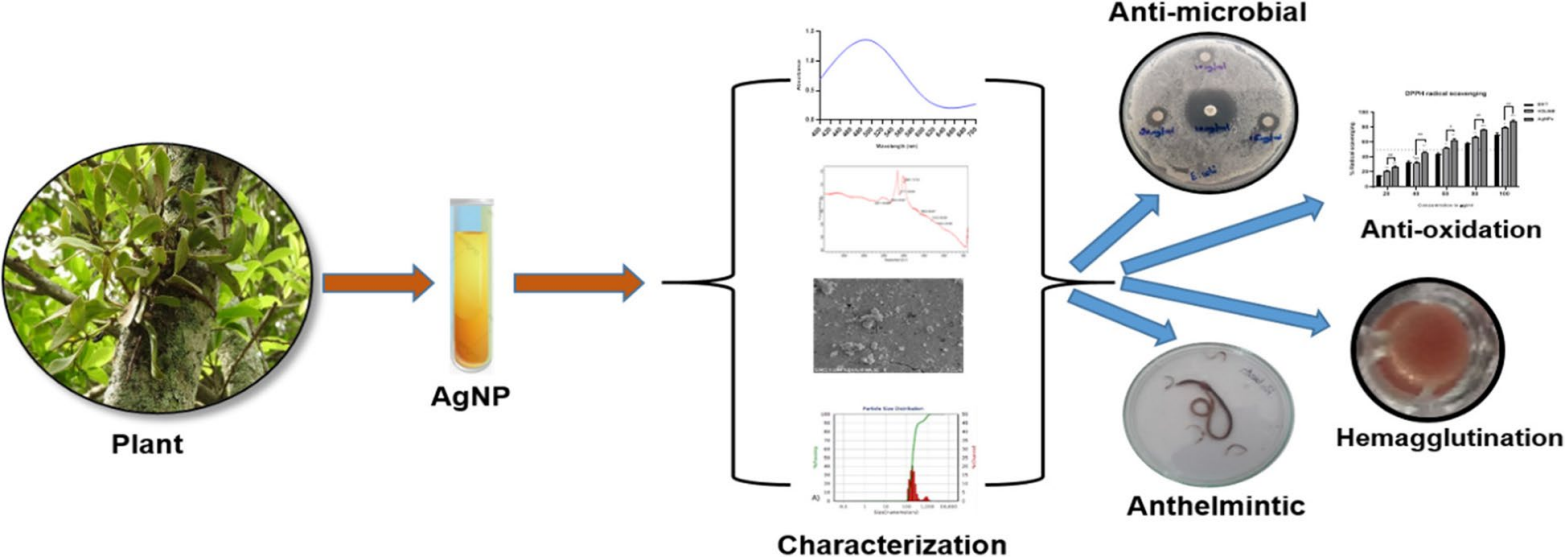
Graphical abstract of the present study

**Fig. 2:**
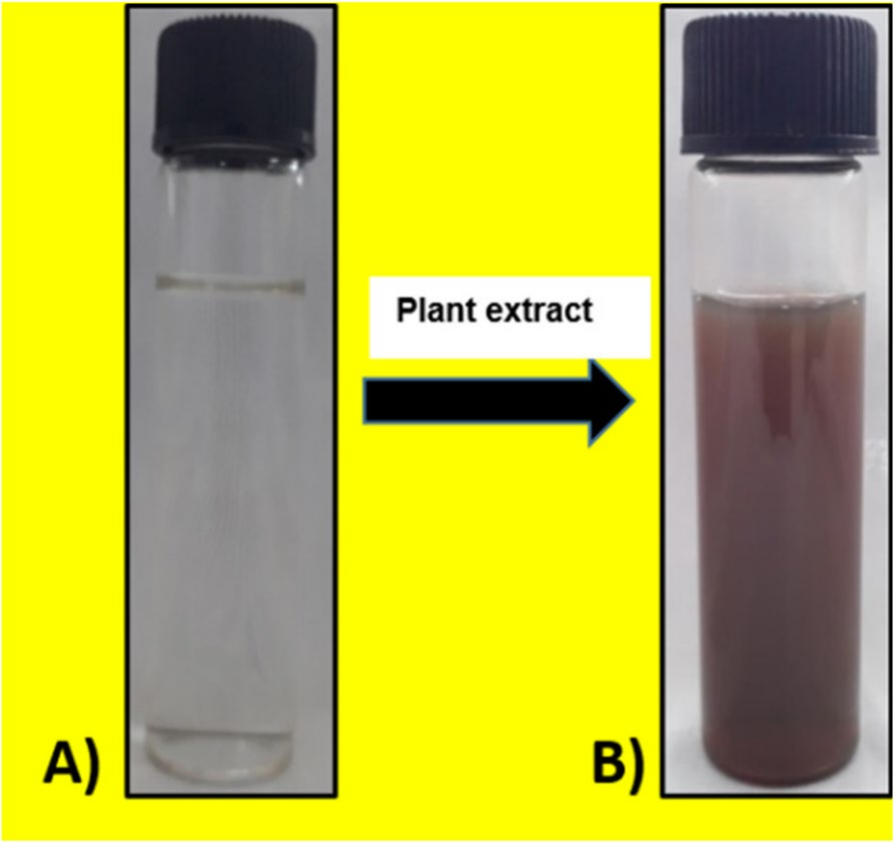
Bioreduction of silver nitrate using VOLWE. (**A**) Before synthesis of AgNPs (**B**) after synthesis of AgNPs

Visual observation of a change in color from pale green to dark brown indicated the formation of silver nanoparticles through bio-reduction of silver nitrate by VOLWE phytochemicals (Fig. 1). The color change was observed at regular intervals, however the change occurred after 3-4 h. Surface plasmon resonance, a size-dependent feature of NPs, is responsible for the dark color. Noticeable color discrimination presents the highest bio-reduction of silver nanoparticles yielding good quantity of silver nanoparticles at a time span of 2 h on constant stirring (Fig. 2). This approach of green synthesis of nanoparticles is found to be least toxic in comparison with the other method which are commonly followed [5]. The currently presented efficiency of green synthesis of VOLWE was comparable to that previously reported [10, 11, 20].

**Fig. 3:**
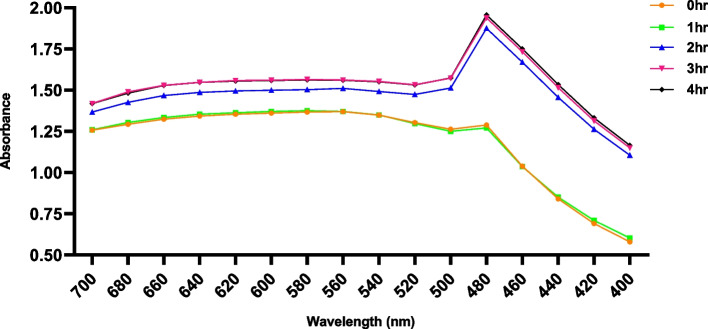
Intensity of AgNPs synthesis on varied stirring time

On time series analysis of silver nanoparticle synthesis using UV–vis spectroscopy on scanning peak between 390–500 nm by UV–visible spectroscope (Figs. 2 and 3), which is a characteristic feature of silver nanoparticles [21, 22].

### Characterization of synthesized nanoparticle

#### UV-spectral analysis of synthesized silver nanoparticles

**Fig. 4:**
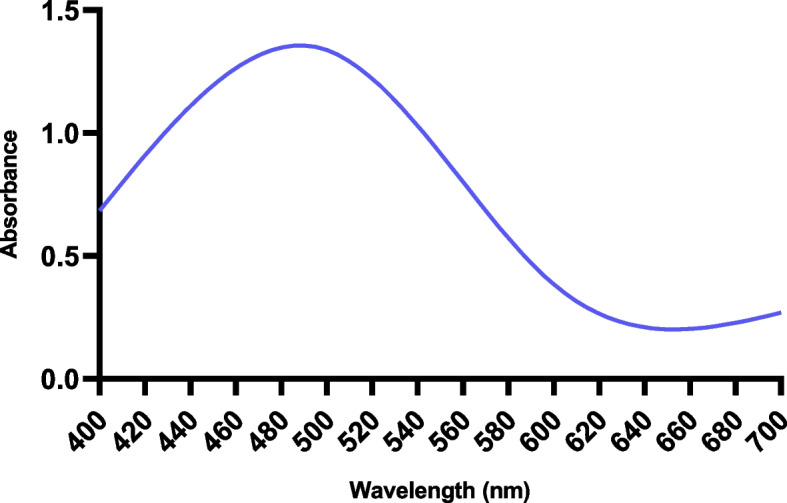
UV–Vis spectra of synthesized AgNP

The importance of AgNO_3_ as well as the presence of components in the leaves for the creation of silver nanoparticles is demonstrated by the UV–Vis spectrum. During the spectral analysis of the synthesized nanoparticles, maximum absorption was observed at 480 nm which is a characteristic surface plasmon resonance peak of green synthesized nanoparticle (Fig. 4) [23]. The results are similar to the previously reported AgNPs [24, 25]. As the absorbance intensity of AgNPs decrease as the concentration of the leaf extract increases [26]. The major peak at 480 nm presented the yield of 30 mg/15 ml of extract.

#### FT-IR spectrum of Synthesized silver nanoparticles

**Fig. 5:**
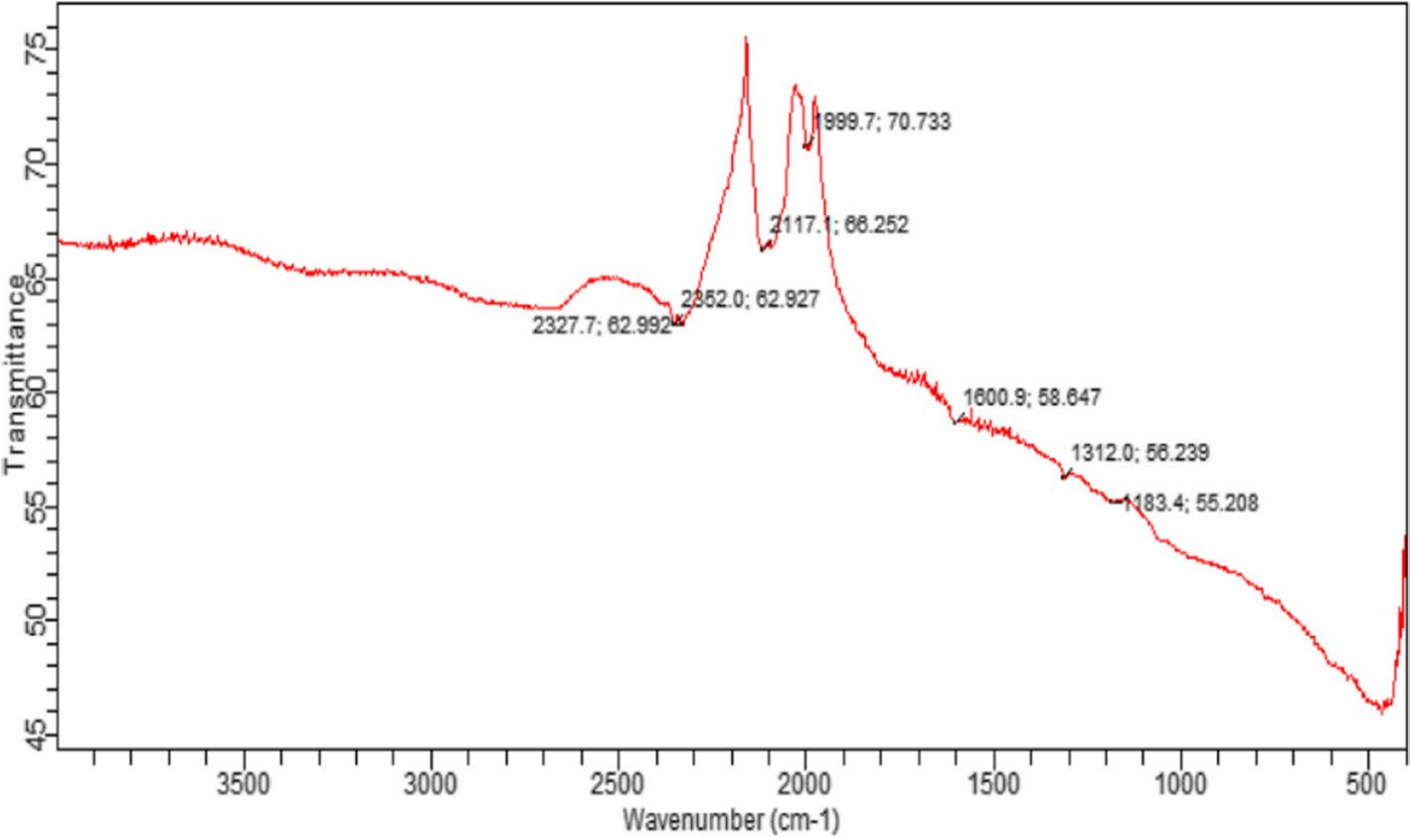
Showing FTIR spectrum of AgNPs

FT-IR analysis of the biosynthesized silver nanoparticles revealed clear absorption peaks at 2327, 2352, 2117, 1999, 1600, 1312, and 1183 cm^−1^ (Fig. 5). The different absorption peaks, such as those at 1000–1300 cm^−1^, which could be aldehyde or ketone, and 1999 cm^−1^, which could be aromatics, and peak at 2300 cm^−1^, which could be the amide I bond of proteins, are caused by carbonyl stretching in proteins and by the interaction of AgNPs during biosynthesis, and the secondary structure was unaffected during the reaction with Ag^+^ ions or after binding with Ag nanoparticles. The carbonyl group of amino acid residues has a good binding ability with silver, indicating the formation of covering layers of AgNPs and acting as a capping mediator to prevent agglomeration and give the medium strength, according to this study. These findings support the existence of proteins that act as reducing and stabilising agents [27].

#### XRD of Synthesized silver nanoparticles

**Fig. 6:**
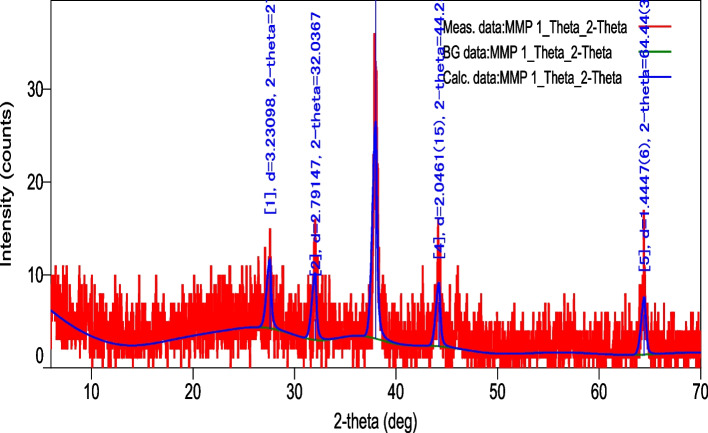
The X-ray diffraction pattern of the biosynthesised AgNPs from the leaf extract

X-ray crystallographic diffraction pattern revealed the crystallinity of bio-reduced AgNPs. The XRD data confirmed the presence of AgNPs. The intensity data were collected over a 2*-theta* range of 10˚-70˚. The strong four peak was located at 3.23˚, 2.79˚, 2.04˚ and 1.44˚. The reflections of metallic silver were revealed by a prominent and high diffraction peak centred at 3.23. The strong peaks clearly show that the synthesised AgNPs have a face-centered cubic (fcc) shape in nature. These results are as per the previous XRD results of biosynthesized AgNPs and shown in Fig. 6.

#### EDAX of synthesized silver nanoparticles

**Fig. 7:**
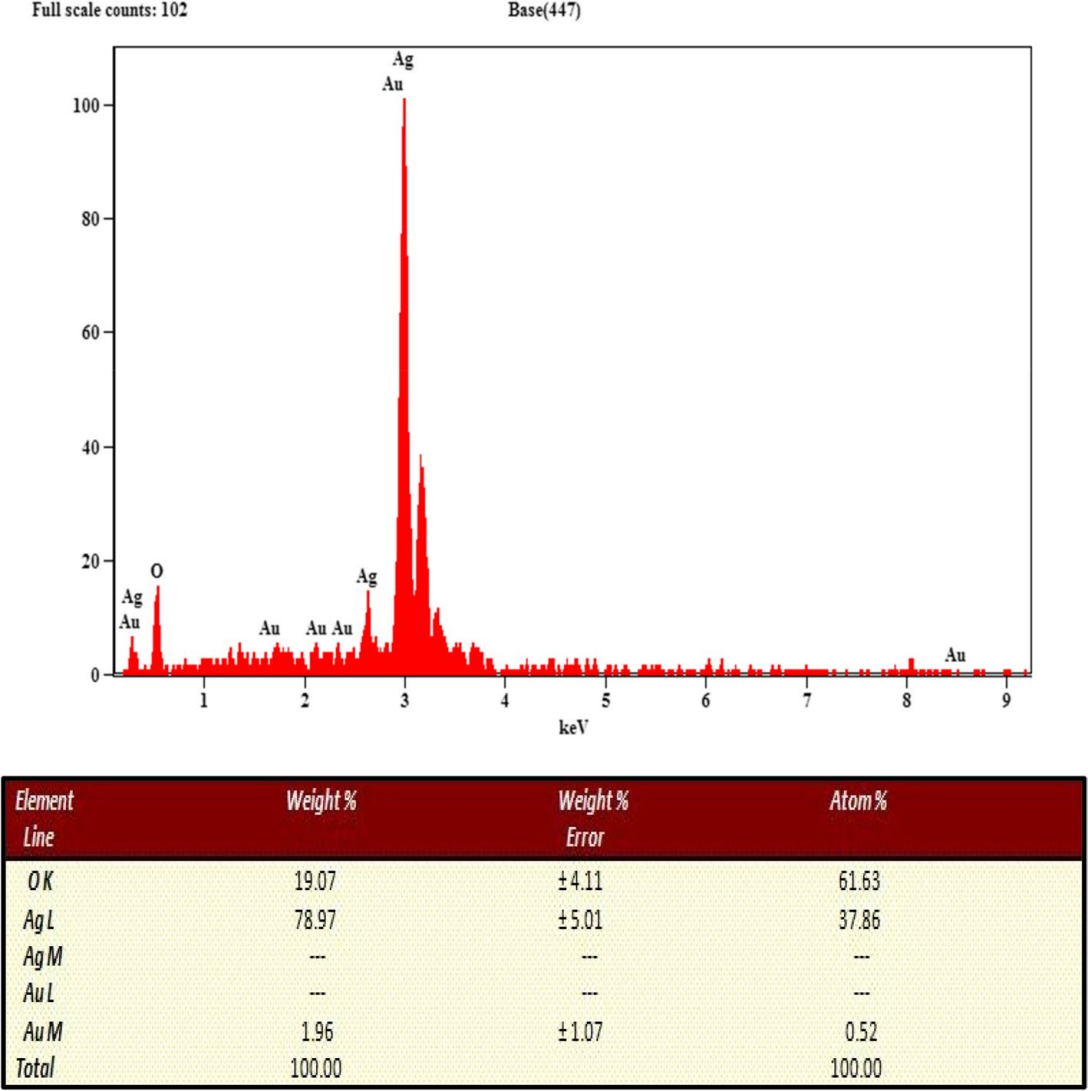
The X-ray diffraction pattern of the biosynthesised AgNPs from the leaf extract

The energy dispersive spectrum of the produced nanoparticles is shown in Fig. 7, indicating the presence of silver as a component element. Due to surface plasmon resonance, metallic silver nanoparticles often have a significant signal peak at 3 keV. EDAX results depicts the quantitative information of biosynthesized AgNPs. The presence of elements such as Ag, Au and O are shown in the inset of Fig. 7. One of the advantages of nanoparticles made with plant extracts over those made with chemical processes is this. The produced silver nanoparticles demonstrate high absorption in the 2.8–3.8 keV region in this study. Jagtap and Bapat and Vijaykumar obtained similar results using *Artemisia nilagirica* leaf and *Artocarpus heterophyllus* seed extracts, with silver nanoparticle production in the 2–4 keV range [11].

#### DLS and zeta potential of Synthesized AgNPs

**Fig. 8:**
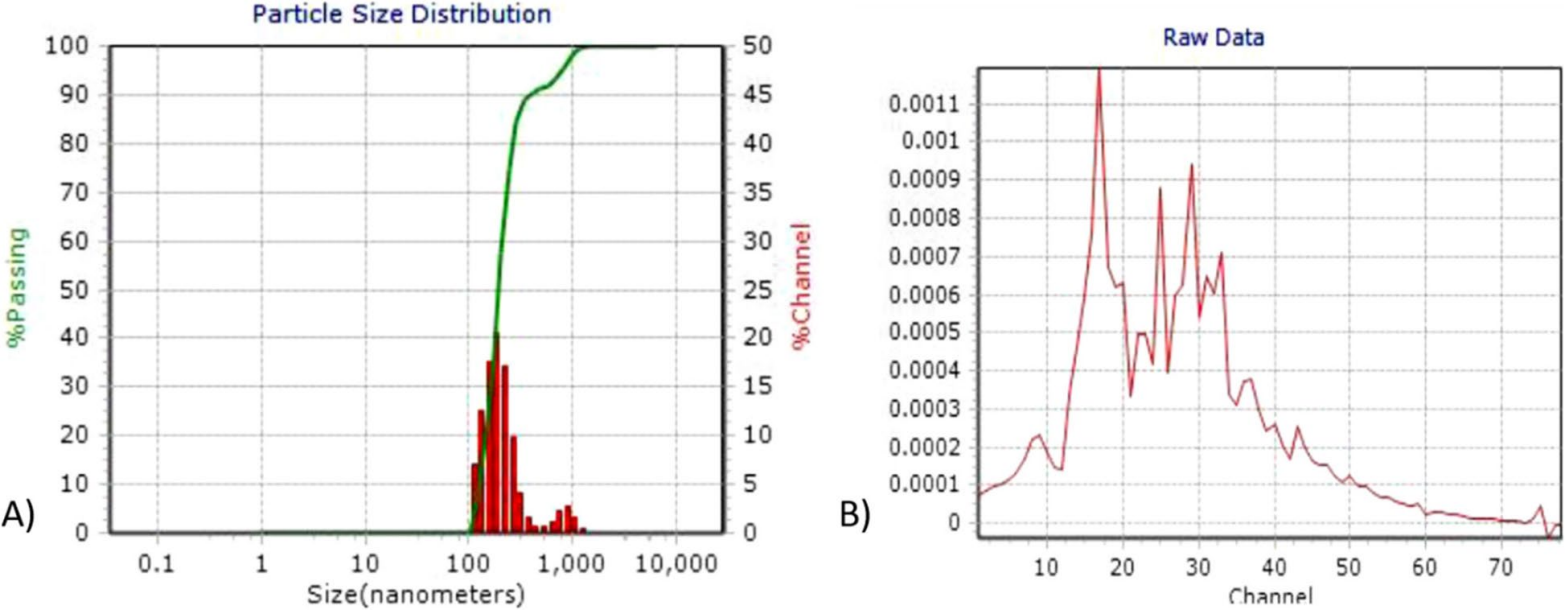
**A**) DLS and **B**) Zeta potential of synthesized nanoparticle

Figure 8A depicts the DLS size distribution image of biosynthesized silver nanoparticles. AgNPs have a size distribution that runs from 105 to 120 nm. When compared to the sharp SPR peak (480 nm) obtained in the UV–Vis spectra, the DLS analyzer's broad spectrum reveals that the particle size is smaller. The particles' average sizes were previously reported to be 53.2 nm. The biosynthesized AgNPs' zeta potential was found to be a strong peak at -17.5 mV. (Fig. 8B). It's thought that the nanoparticles' surfaces are negatively charged and diffused in the liquid. The negative value validates the particles' repulsion and demonstrates their stability [11].

#### SEM analysis of synthesized AgNPs

**Fig. 9:**
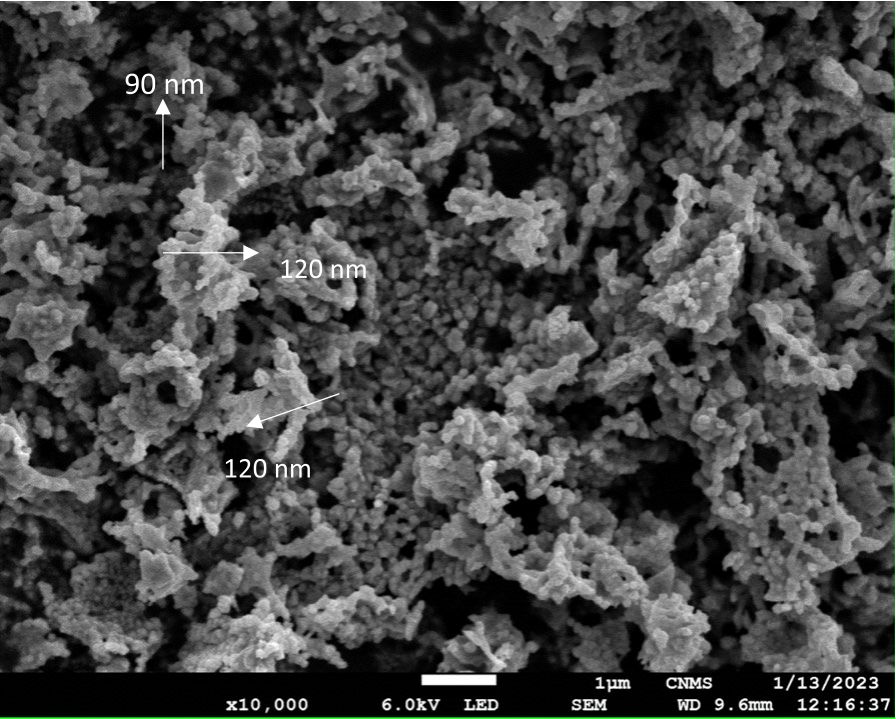
SEM analysis of synthesized AgNPs

SEM analysis was used to examine the surface morphology and topography of bio-synthesised silver nanostructures. The AgNPs made with VOLWE have well-defined nanoscale architectures with apparent spherical morphologies ranging from around 90, 120, and 200 nm (Fig. 9).

#### TEM analysis of synthesized AgNPs

**Fig. 10:**
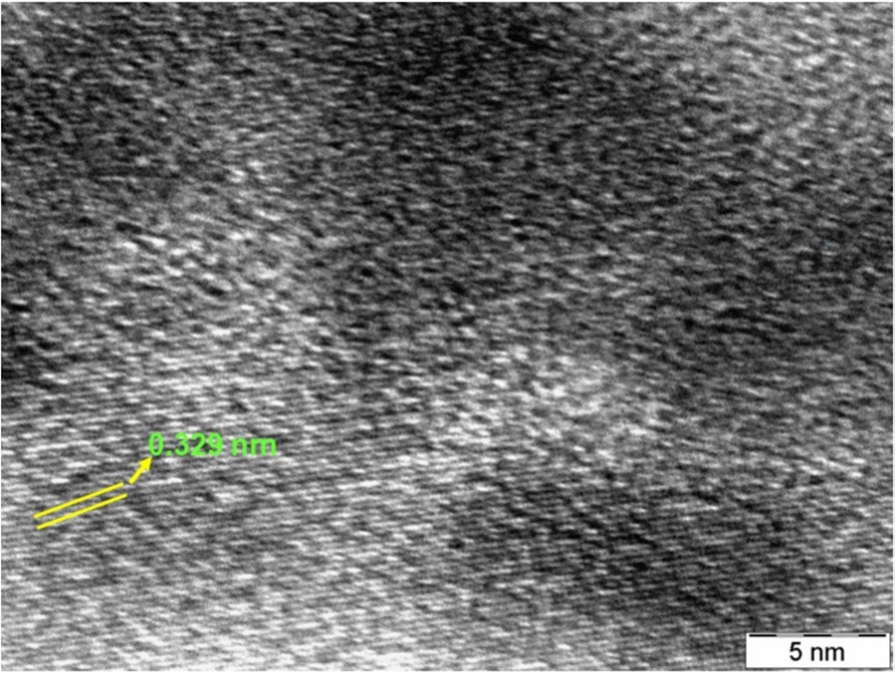
TEM analysis of synthesized AgNPs

The morphology and the size of the as-prepared AgNPs was investigated by High Resolution Transmission Electron Microscopy (HR-TEM) analysis. Although, literature reports mention the various other morphology of AgNPs synthesized using a similar method [23], herein, the as-prepared AgNPs is typically spherical in shape with an average particle size of 22 nm. (Fig. 10). As depicted in Fig. 10, the lattice fringes having inter planar distance of 0.329 nm, which is assigned to (111) plane.

### Anti-oxidant activity of AgNPs

#### DPPH

**Fig. 11:**
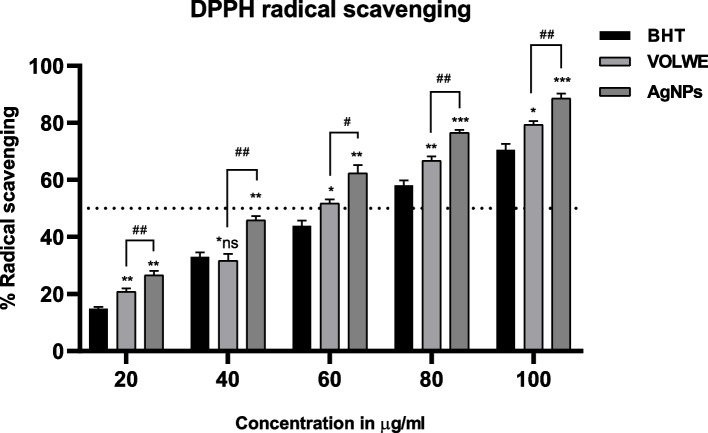
Effect of AgNPs on DPPH radical scavenging. Statistical significance was calculated using two way ANOVA with Turkey’s multiple comparison test. * indicates comparison with BHT. # indicates comparison between extract and AgNPs

DPPH analysis was performed to analyze the radical scavenging capacity of AgNPs against chemically generated stable radical [28]. The effective concentration (EC50) of AgNPs was found to be very efficient (52.34 µg/ml) in comparison with the standard drug BHT (53.84 µg/ml) where as VOLWE showed EC50 at 57.60 µg/ml. Statistical analysis showed a significant increase in the radical scavenging capacity of the AgNPs than VOLWE and BHT (Fig. 11) and reconfirms the efficiency of AgNPs radical scavenging ability. Similar results are reported previously by SNP from different plant sources [29–31].

#### Radical power capacity

**Fig. 12:**
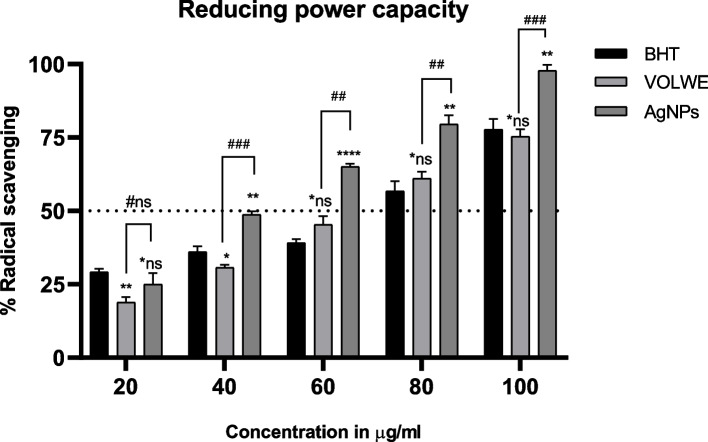
Effect of AgNPs on reducing power capacity. Statistical significance was calculated using two way ANOVA with Turkey’s multiple comparison test. * indicates comparison with BHT. # indicates comparison between extract and AgNPs

AgNPs not only had higher free radical scavenging properties than BHT, but also had higher reducing power, with a percentage increase in absorbance directly proportional to reducing power of up to 95%, whereas ascorbic acid had a percentage increase in absorbance of only 75%, which is significantly lower than AgNPs at 100 g/ml concentration (Fig. 12). The effective concentration (EC50) of AgNPs was found to be very efficient (53.42 µg/ml) in comparison with the standard drug BHT (74.27 µg/ml) where as VOLWE showed EC50 at 59.52 µg/ml. Comparable work has been conducted previously in the using different extract [30, 32]

#### Nitric oxide radical scavenging

**Fig. 13:**
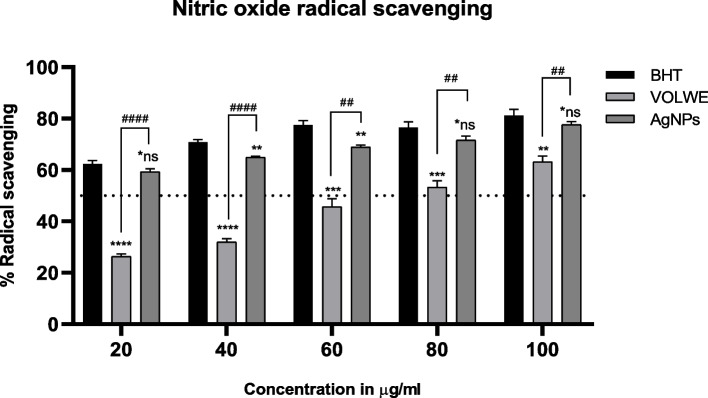
Effect of AgNPs on nitric oxide radical scavenging. Statistical significance was calculated using two way ANOVA with Turkey’s multiple comparison test. * indicates comparison with BHT. # indicates comparison between extract and AgNPs

Nitric oxide (NO) is a sever disease marker in the human body. NO scavenging activity of AgNPs would be by inhibiting the formation of nitrite through direct competition with the oxygen radical [33]. The effective concentration (EC50) of AgNPs was found to be very efficient (56.01 µg/ml) in comparison with the standard drug BHT (43.52 µg/ml) where as VOLWE showed EC50 at 59.30 µg/ml. On comparison with the other radical scavenging ability of AgNPs, NO scavenging ability is almost similar to the standard at higher concentration with no significant difference (Fig. 13). Similar reports are stated in previous studies [32–34].

### Antimicrobial activity of AgNPs

**Fig. 14:**
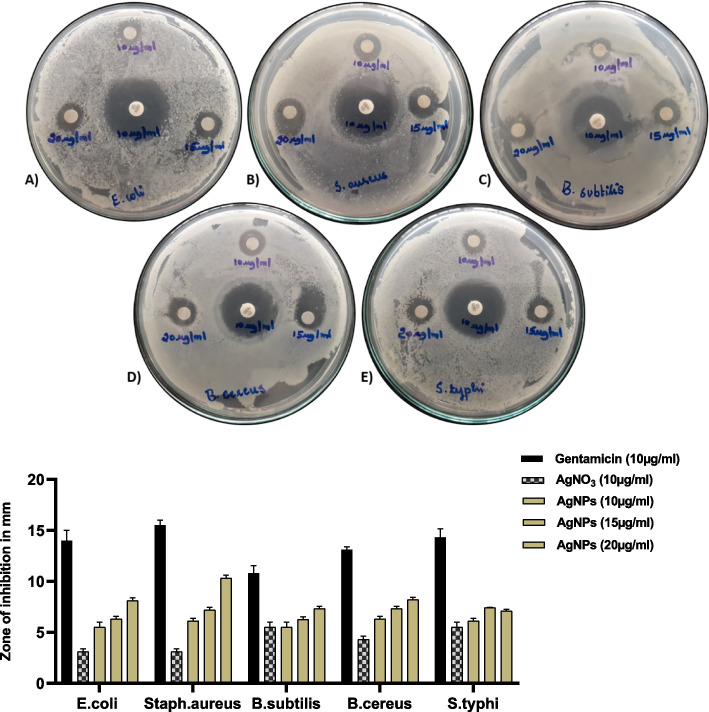
Antibacterial activity of AgNPs against different bacteria. **A**) *E.coli* **B**) *Staph.aureus c*) *B.subtilis* **D**) *B.cereus* E) *S.typhi*

SNPs showed prominent anti-bacterial property in concentration dependent manner against *E.coli, Staph.aureus, B.subtilis, B.cereus and S.typhi* on comparison with the standard drug gentamicin (10 µg/ml). Highest zone of inhibition was found in case of *Staph.aureus* (10.3 ± 0.3) followed by *B.cereus* (8.2 ± 0.25), *E.coli* (8.1 ± 0.28), *B.subtilis* (7.3 ± 0.26) and *S.typhi* (7.1 ± 0.15). These results were found to be in accordance with the previous reports [35] (Fig. 14).

Peptidoglycan is a thick layer of the bacterial cell wall that is built up of linear polysaccharide chains joined by short peptides, resulting in a stiffer structure that makes AgNP penetration difficult. The silver cations produced by AgNPs, which act as reservoirs for the Ag bactericidal agent, are undoubtedly responsible for the strong bactericidal activity. AgNPs were widely used in antimicrobial coatings in medical instrument production and food packaging as a result of this discovery. Despite the fact that both silver nanoparticles and silver nitrate suppressed the growth of all gram-positive and gram-negative bacteria, silver nanoparticles outperformed silver nitrate in antibacterial activity [20, 36–38]. It is universally known that the bioactives exhibit antimicrobial activity by acting as antioxidants. Our confirmation of antioxidant nature of the nanoparticles are suggestive of the mode of action to casuse antimicrobial effect. Furthermore studies, are in progress to decipher the mechanism of action. (https://www.frontiersin.org/articles/10.3389/fmicb.2019.00829/full).

### Anthelmintic activity

Adult motility *in-vitro* assay was conducted on mature live earthworms. The assay was performed on adult earthworms. Because of its morphological and physiological similarities to the human intestinal round worm parasite. Earthworms have been utilised widely for preliminary in-vitro screening of anthelminthic substances in-vitro due to their ease of availability [39].

**Fig. 15:**
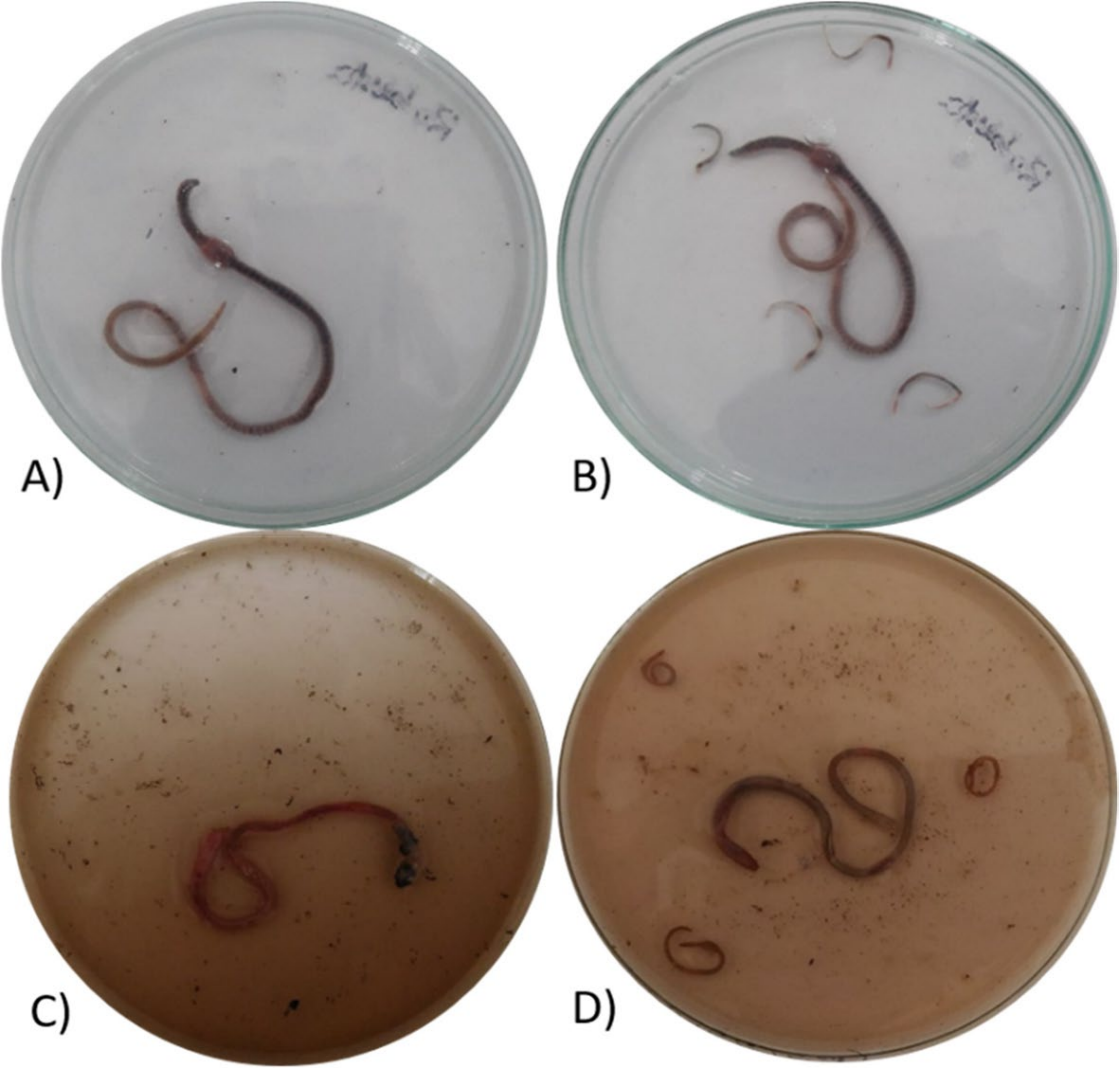
Effect on earthworm. **A**) Saline **B**) AgNO_3_ **C**) VOLWE (100 µg/ml)** D**) AgNPs

AgNPs had great impact on the paralysis and death time on treatment. Comparatively with the VOLWE treatment, the AgNPs took very less time to paralyze the earthworm leading to death 5.4 ± 0.3 and 6.5 ± 0.6 respectively (Fig. 15, Table 3).

**Table 3:** Anthelmenthic activity of the AgNPs. Data presented mean ± SEM. Observation was done up to 120 min

Constituents	Paralysis time (min)	Death time (min)
**Saline**	> 120	> 120
**AgNO**_**3**_	36.9 ± 0.2	66.3 ± 0.4
**VOLWE**	22.7 ± 0.7	40.5 ± 0.7
**AgNPs**	5.4 ± 0.3	6.5 ± 0.6

### Hemagglutination activity

**Fig. 16:**
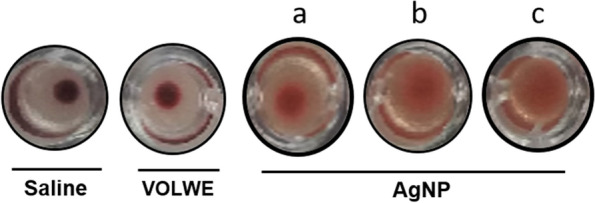
Hemagglutination effect of AgNPs. **a**) 60 µg/ml AgNP **b**) 80 µg/ml AgNP** c**) 100 µg/ml AgNP

To find the biocompatibility of the AgNPs in the biological system, its ability of hemagglutination was performed using human blood. Figure 16 showed hemagglutination property of the AgNPs above 80µg/ml concentration, below which it dint show any agglutination effect. This interprets the biocompatibility nature of the AgNPs using the water extract. Similar effects was shown in previous reports of plant extracts with silver nanoparticles [27]. Hence the presently studied SNPs can be an effective drug in the invivo models.

## Conclusion

In the present study, unexplored parasitic plant was chosen with medicinal properties. The plant was known for its traditional medicinal uses along with its significant toxic nature. Silver nanoparticles were synthesised with the water extract of the plant which eventually reduce its toxic effects mainly because of its size. On characterization of the AgNPs, confirmed its nanometer size for further evaluation. Anti-bacterial effect of AgNps were also significant in inhibiting the potent bacterial growth. AgNPs ingrained its effectiveness in radical scavenging using stable and unstable free radicals in dose dependent manner. Anhelmintic consequence of AgNPs was substantial. The biocompatibility AgNPs by hemagglutination property was evident. Overall the study boosts the biologically useful potency of the AgNPs. Furthermore, studies are being carried out to selectively use the functionalized AgNPs based on their size, and also decipher their mechanism of action enabled by their antioxidant nature.

## Supplementary Information

**Additional file 1. **

## Data Availability

The datasets used and/or analysed during the current study are available from the corresponding author on reasonable request.

## Abbreviations

SNPs: Synthesized nanoparticles
AgNPs: Silver nanoparticles
FT-IR: Fourier-transform infrared spectroscopy
DLS: Dynamic Light Scattering
XRD: X-ray diffraction
EDAX: Energy-dispersive X-ray spectroscopy
DPPH: 1,1-Diphenyl-2-picryl-hydrazyl
