# Supplementary material for: Assessment of antimicrobial and anthelmintic activity of silver nanoparticles bio-synthesized from Viscum orientale leaf extract
Source: BMC Complement Med Ther. 2023 May 22;23:167. doi: 10.1186/s12906-023-03982-1 (PMC10204336; doi:10.1186/s12906-023-03982-1)
Supplement: Supplementary file 1 — Additional file 1. [file 12906_2023_3982_MOESM1_ESM.docx]

**RAW data of statistical analysis**

**Nitric oxide radical scavenging**

| **Comparatives** | **Mean** | **SEM** | **p-value** |
| --- | --- | --- | --- |
| 20μg/ml |  |  |  |
| BHT vs. Extract | 35.97 | 0.9621 | <0.0001 |
| BHT vs. AgNPs | 3.057 | 1.016 | 0.0845 |
| Extract vs. AgNPs | -32.91 | 0.8801 | <0.0001 |
|  |  |  |  |
| 40μg/ml |  |  |  |
| BHT vs. Extract | 38.83 | 1.002 | <0.0001 |
| BHT vs. AgNPs | 5.920 | 0.6730 | 0.0092 |
| Extract vs. AgNPs | -32.91 | 0.8306 | <0.0001 |
|  |  |  |  |
| 60μg/ml |  |  |  |
| BHT vs. Extract | 31.79 | 2.022 | 0.0009 |
| BHT vs. AgNPs | 8.620 | 1.098 | 0.0097 |
| Extract vs. AgNPs | -23.17 | 1.831 | 0.0068 |
|  |  |  |  |
| 80μg/ml |  |  |  |
| BHT vs. Extract | 23.21 | 1.890 | 0.0006 |
| BHT vs. AgNPs | 4.887 | 1.516 | 0.0758 |
| Extract vs. AgNPs | -18.33 | 1.688 | 0.0022 |
|  |  |  |  |
| 100μg/ml |  |  |  |
| BHT vs. Extract | 17.98 | 1.843 | 0.0014 |
| BHT vs. AgNPs | 3.587 | 1.531 | 0.1934 |
| Extract vs. AgNPs | -14.40 | 1.420 | 0.0035 |

| IC50 | 43.52 | 59.30 | 56.01 |
| --- | --- | --- | --- |

**Reducing power**

| **Comparatives** | **Mean** | **SEM** | **p-value** |
| --- | --- | --- | --- |
| 20μg/ml |  |  |  |
| BHT vs. Extract | 10.37 | 1.190 | 0.0044 |
| BHT vs. AgNPs | 4.197 | 2.252 | 0.3231 |
| Extract vs. AgNPs | -6.170 | 2.388 | 0.1639 |
|  |  |  |  |
| 40μg/ml |  |  |  |
| BHT vs. Extract | 5.370 | 1.222 | 0.0453 |
| BHT vs. AgNPs | -12.66 | 1.279 | 0.0030 |
| Extract vs. AgNPs | -18.03 | 0.8628 | 0.0001 |
|  |  |  |  |
| 60μg/ml |  |  |  |
| BHT vs. Extract | -6.237 | 1.804 | 0.0895 |
| BHT vs. AgNPs | -25.90 | 0.9518 | <0.0001 |
| Extract vs. AgNPs | -19.67 | 1.756 | 0.0063 |
|  |  |  |  |
| 80μg/ml |  |  |  |
| BHT vs. Extract | -4.350 | 2.384 | 0.2850 |
| BHT vs. AgNPs | -22.85 | 2.664 | 0.0023 |
| Extract vs. AgNPs | -18.50 | 2.251 | 0.0036 |
|  |  |  |  |
| 100μg/ml |  |  |  |
| BHT vs. Extract | 2.363 | 2.483 | 0.6440 |
| BHT vs. AgNPs | -20.10 | 2.318 | 0.0056 |
| Extract vs. AgNPs | -22.47 | 1.836 | 0.0008 |

| IC50 | 74.27 | 59.52 | 53.42 |
| --- | --- | --- | --- |

| **Sl. no** | **Name of the organism** | **Inhibition zone(mm)** | | | |  |
| --- | --- | --- | --- | --- | --- | --- |
|  |  | **Gentamicin**  **(10µg/ml)** | **AgNO_3_**  **(10µg/ml)** | **AgNPs**  **(10µg/ml)** | **AgNPs**  **(15µg/ml)** | **AgNPs**  **(20µg/ml)** |
| **1** | ***E.coli*** | 14±1 | 3.1±0.28 | 5.5±0.5 | 6.3±0.28 | 8.1±0.28 |
| **2** | ***Staph.aureus*** | 15.5±0.5 | 3.1±0.28 | 6.1±0.28 | 7.2±0.25 | 10.3±0.3 |
| **3** | ***B.subtilis*** | 10.8±0.76 | 5.5±0.5 | 5.5±0.5 | 6.26±0.25 | 7.3±0.26 |
| **4** | ***B.cereus*** | 13.1±0.28 | 4.3±0.32 | 6.3±0.28 | 7.3±0.26 | 8.2±0.25 |
| **5** | ***S.typhi*** | 14.3±0.86 | 5.5±0.5 | 6.1±0.28 | 7.4±0.05 | 7.1±0.15 |

**DPPH radical scavenging**

| **Comparatives** | **Mean** | **SEM** | **p-value** |
| --- | --- | --- | --- |
| 20μg/ml |  |  |  |
| BHT vs. Extract | -5.867 | 0.7424 | 0.0099 |
| BHT vs. AgNPs | -11.73 | 0.8969 | 0.0040 |
| Extract vs. AgNPs | -5.867 | 1.072 | 0.0134 |
|  |  |  |  |
| 40μg/ml |  |  |  |
| BHT vs. Extract | 1.307 | 1.616 | 0.7206 |
| BHT vs. AgNPs | -12.83 | 1.242 | 0.0011 |
| Extract vs. AgNPs | -14.13 | 1.576 | 0.0038 |
|  |  |  |  |
| 60μg/ml |  |  |  |
| BHT vs. Extract | -7.967 | 1.359 | 0.0119 |
| BHT vs. AgNPs | -18.57 | 1.948 | 0.0026 |
| Extract vs. AgNPs | -10.60 | 1.798 | 0.0209 |
|  |  |  |  |
| 80μg/ml |  |  |  |
| BHT vs. Extract | -8.687 | 1.344 | 0.0071 |
| BHT vs. AgNPs | -18.45 | 1.204 | 0.0008 |
| Extract vs. AgNPs | -9.767 | 1.067 | 0.0026 |
|  |  |  |  |
| 100μg/ml |  |  |  |
| BHT vs. Extract | -8.867 | 1.403 | 0.0135 |
| BHT vs. AgNPs | -18.00 | 1.563 | 0.0009 |
| Extract vs. AgNPs | -9.133 | 1.213 | 0.0053 |

| IC50 | 53.84 | 57.60 | 52.34 |
| --- | --- | --- | --- |
|  | BHT | Extract | AgNp |
|  |  |  |  |
